# Rapid healing of a patient with dramatic subacute combined degeneration of spinal cord: a case report

**DOI:** 10.1186/s13104-016-2344-4

**Published:** 2017-01-03

**Authors:** Florian C. Roessler, Stephanie Wolff

**Affiliations:** 1 Department of Neurology, Justus-Liebig-University Giessen, Klinikstraße 33, 35385 Gießen, Germany; 2 Klinik und Poliklinik für Neurologie, Universitätsklinikum Standort Gießen, Klinikstraße 33, 35385 Gießen, Germany

**Keywords:** Subacute combined degeneration of spinal cord, Cobalamin, Methylmalonic acid, Holotranscobalamin, Homocysteine, Autoimmune gastritis

## Abstract

**Background:**

Prevalence of cobalamin deficiency is high especially in older patients and an immediate therapy start is necessary to prevent irreversible neurological damages. Unfortunately, the diagnosis of cobalamin deficiency is difficult and at present, there is no consensus for diagnosis of this deficiency. Therefore, we aim to elucidate a meaningful diagnostic pathway by a case report with an initially misleading medical history.

**Case presentation:**

A 57 year-old Caucasian man suffering from dramatic myelosis of the cervical posterior columns. Apart from associated neurological symptoms (tactile hypaesthesia, reduced vibration sensation, loss of stereognosis and of two-point-discrimination) there were no further complaints; especially no gastrointestinal, haematological or psychiatric disorders were provable. Cobalamin (vitamin B12) serum level was normal. The diagnosis of subacute combined degeneration of spinal cord was confirmed by an elevated methylmalonic acid, and hyperhomocysteinemia. Cobalamin deficiency was caused by asymptomatic chronic atrophic inflammation of the stomach with a lack of intrinsic factor producing gland cells. This was revealed by increased gastrin and parietal cell antibodies and finally confirmed by gastroscopy. Parenteral substitution of cobalamin rapidly initiated regeneration.

**Conclusions:**

This case demonstrates that normal cobalamin serum levels do not rule out a cobalamin deficiency. In contrast, path-breaking results can be achieved by determining homocysteine, holotranscobalamin, and methylmalonic acid.

## Background

Prevalence of cobalamin deficiency in general population is about 4% [1]. In older patients (>65 years) functional cobalamin deficiency was found in 10–30% of all cases [2, 3]. Frequently, the diagnosis of cobalamin deficiency is difficult, because anaemia or macrocytosis are frequently absent, cobalamin concentrations are mostly borderline [4], and solely psychiatric syndromes are present which are sometimes variable, unspecific, subtle, and uneven in rate [5, 6]. Therefore, the exact prevalence of clinically significant cobalamin deficiency is not known [7]. Early diagnostic and an immediate therapy start are necessary to prevent irreversible neurological damages [8]. However, at present, there is no consensus or guideline for the diagnosis of this deficiency.

Humans are not able to synthesize cobalamin. Food of animal source is the only natural source of cobalamin in human diet. In the stomach, ingested cobalamin is detached from its protein-binding by pepsin and hydrochloric acid. Then, it is bound to the glycoproteins haptocorrin and intrinsic factor (IF) secreted by gastric mucosa. The majority of the required cobalamin uptake takes place in the terminal ileum by binding of the cobalamin-IF-complex to receptors of the mucosa cells [9]. Inside the enterocytes cobalamin is released and bound to its carrier protein transcobalamin II. Thereby, holotranscobalamin (holoTC) originates. In this way cobalamin circulates in the blood and gets absorbed by body cells. In the cytoplasm, the released cobalamin is converted into methylcobalamin. In the mitochondria, cobalamin is converted into adenosylcobalamin.

Methylcobalamin and folates are co-factors in the methionine-synthase mediated conversion of homocysteine to methionine, which is essential for nucleotide synthesis and genomic and non-genomic methylation [5]. Therefore, a lack of methylcobalamin leads to a disturbed cell multiplication. Homocysteine accumulates at the same time. High concentrations of homocysteine are associated with an increased cardiovascular risk [10–13]. Furthermore, homocysteine seems to have neurotoxic properties causing vascular dementia and Alzheimer's disease [14, 15]. Adenosylcobalamin is a co-factor for methylmalonyl-CoA mutase converting methylmalonyl-CoA to succinyl-CoA. Succinyl-CoA plays a decisive role in the citric acid cycle. Therefore, a lack of adenosylcobalamin disrupts the proliferation, maturation, and regeneration of neurons and leads to an accumulation of methylmalonic acid (MMA).

Clinically, cobalamin deficiency is manifested mainly by haematological and neuropsychiatric symptoms. Frequently, these symptoms arise before the lower limit value of cobalamin is reached [16]. In contrast, macrocytosis evolves later. Subacute combined degeneration of spinal cord (SACD) is a frequent consequence of cobalamin deficiency. In most cases this disease is restricted to the posterior columns of the upper cervical and thoracic segments associated with tactile sensibility loss and proprioceptive problems [4, 17, 18]. White matter damages comply with an abnormal myelination [19] probably caused by (1) reduced methyl group availability resulting from a lack of methylcobalamin [17, 20] and (2) nonphysiological fatty acids toxicity resulting from a decreased activity of the adenosylcobalamin dependent methylmalonyl-CoA mutase [20]. Focal gliosis results from homocysteine-induced toxicity to the endothelium [20]. Subsequently, a less extended demyelination of the spinocerebellar tracts and also an involvement of the lateral columns and pyramidal tracts can be seen, which typically gets started in the thoracic cord, but can extend to involve other levels [17]. This might lead to ataxia, paresis, hyperreflexia, and bladder dysfunction. Later, peripheral nerves, cerebrum, and in rare cases also optic nerves are damaged [17]. Additionally, patients may become depressed or suffer from psychosis [4, 18]. Many patients have a macrocytosis [21], glossitis and cutaneous manifestations like hyperpigmentation, hair and nail changes [22].

Common reasons for cobalamin deficiencies can be divided into four groups (Table 1): diminished supply of cobalamin, disruption of cobalamin processing in the stomach (caused for instance by an autoimmune gastritis resulting from antibodies reacting with parietal cells leading to a decreased emission of intrinsic factor), intestinal resorption problems, and defective transport and intracellular metabolism [21, 23, 24].

**Table 1:** Common reasons for cobalamin deficiencies

Diminished supply	
Vegan nutrition	
Alcohol abuse	
Parasitic infections (e.g. fish tapeworm)	
Reduced food intake (older people)	
Pregnancy (relative deficit)	
Disruption of cobalamin processing in the stomach	
Gastric bypass/post-gastrectomy	
Chronic gastritis (e.g. induced by alcohol abuse, helicobacter pylori infection)	
Autoimmune gastritis resulting from antibodies reacting with parietal cells (pernicious anaemia)	
Proton pump inhibitors and H2-receptor antagonists (sustained release of cobalamin)	
Metformin, cytostatics, methyldopa, aminoglycosides e.g. (medicinal side effect)	
Intestinal resorption problems	
Intestinal bypass/ileal resection	
Pathogenic intestinal flora	
Ulcerating colitis	
Crohn’s disease	
Zollinger-Ellison syndrome	
Imerslund-Gräsbeck syndrome	
Defective transport and intracellular metabolism	
Congenital deficiency in transcobalamin II	
Congenital deficiency in various intracellular enzymes	

**Fig. 1:**
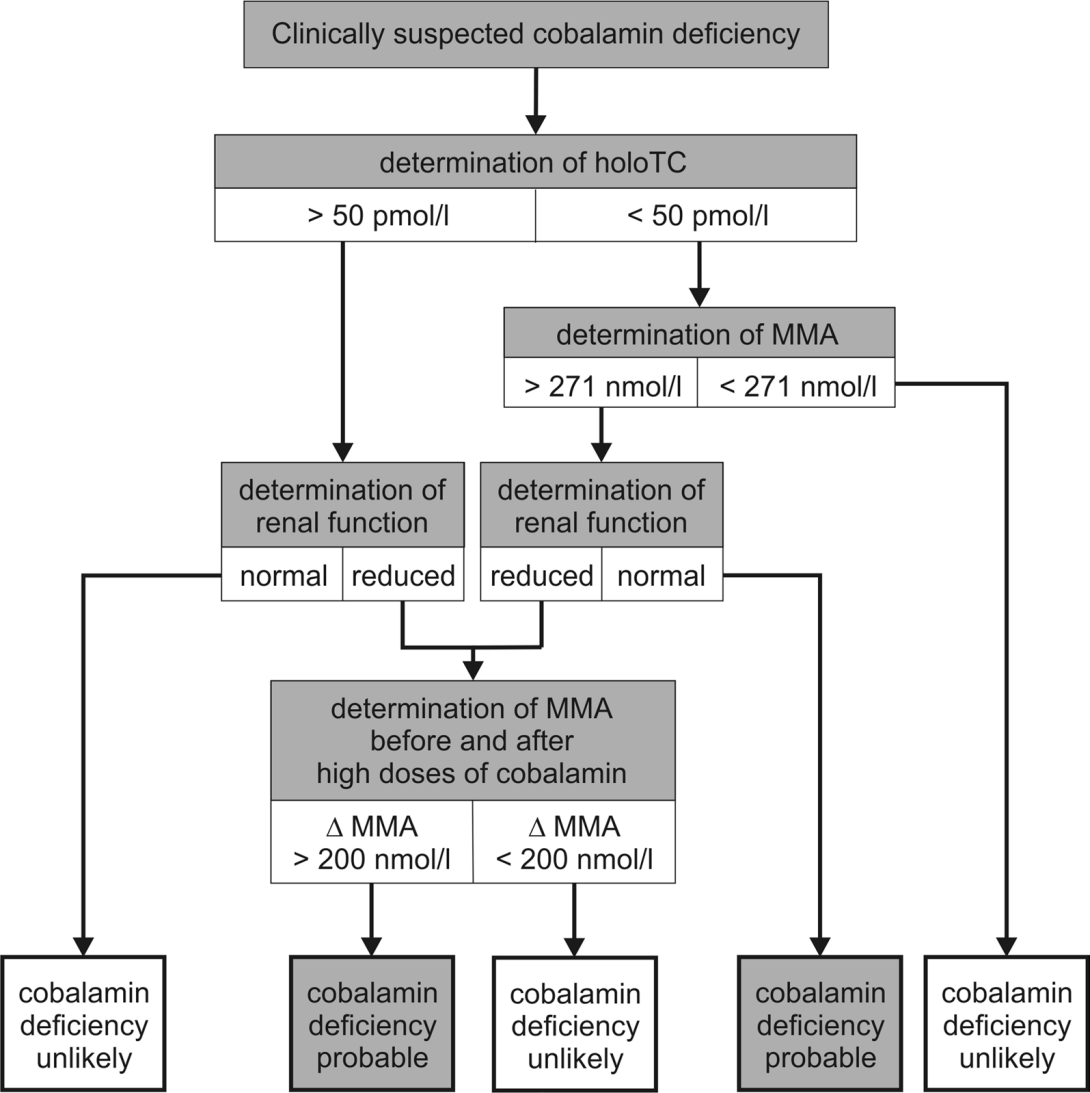
Diagnostic pathway to prove cobalamin deficiency. Up to now, no consensus exists about the best diagnostic pathway to prove cobalamin deficiency [7]. This pathway is a modification of recommendations made by Herrmann et al. [7].* MMA* methylmalonic acid,* holoTC* holotranscobalamin. Limit values for MMA and holoTC specified here are in accordance with those of other authors [7, 24, 29, 32]. For follow-up we recommend the measurement of homocysteine (normal: 5.0–15.0 µmol/l; pathological threshold: >25 µmol/l) [28]

Finally, cobalamin deficiency with inconspicuous blood values for cobalamin and holoTC is common in patients suffering from kidney diseases [25]. Probably, this is caused by a disrupted cellular absorption of holoTC and by secondary accumulation of holoTC resulting from a disturbed filtration of transcobalamin in the kidneys [26]. This leads to an intracellular lack of cobalamin and to increased values of cobalamin dependent metabolites (MMA and homocysteine). On the other hand, patients suffering from kidney diseases might also have increased values of MMA although no cobalamin deficiency is present [2, 25]. Therefore, Herrmann et al. recommend in cases of renal dysfunction to verify a real cobalamin deficiency by detection of a significant reduction of MMA (ΔMMA > 200 nmol/l) after probatory substitution with cobalamin [7] (Fig. 1).

Clinical improvement and full recovery from myelopathy can occur when substitution of cobalamin and folic acid is started in the early stages of the disease [27].

We would like to present a case, in which medical history and imaging initially pointed to a traumatic or malignant cause of solely neurological complaints. This case illustrates the need of a targeted laboratory diagnostic when clinical examination raises reasonable suspicion of SACD.

## Case presentation

### Medical history

Seven months before presentation, a 57 year-old Caucasian man fell off a two meter high roof, suffering from a left-hand serial rip fracture and a fracture of the processus transversi of the thoracic vertebral bodies 6 and 7. Three months later, he recognized for the first time a sustainable tactile hypaesthesia and paraesthesia beginning in both hands and extending to both shoulders and to the thorax double-sided within the following months. He also described a narrowed sensation within the thorax. Three and a half months after the start of sensibility loss a magnetic resonance tomography of the cervical spine was performed outward. There were no other diseases or allergies and no sustained medication intake. Nutritional status was normal with no restrictive dietary habits.

### Physical examination

The patient suffered from a symmetric hypaesthesia of both arms reaching from the fingers up to the middle of the upper arms and double-sided at the thorax from Th2 to Th10. He had a pathological two-point-discrimination at both arms and at the thorax (he only recognized distances >7 cm) and a disturbed stereognosis: the patient was not able to distinguish a pen from a rolled-up bandage. Additionally, he offered a reduced pallaesthesia: vibration sensibility was reduced to 3–4/8 on both sides of the distal radius, and to 0/8 on both malleoli mediales and to 6/8 on both tibiae. Perception of temperature and pain and sense of position were not affected. Further examination did not reveal any abnormalities. Gait was unremarkable, reflexes were normal: There were no pyramidal tract symptoms, no pareses and no mental abnormalities. The patient was of good general condition with a normal weight. He had no glossitis.

### Diagnostic

**Fig. 2:**
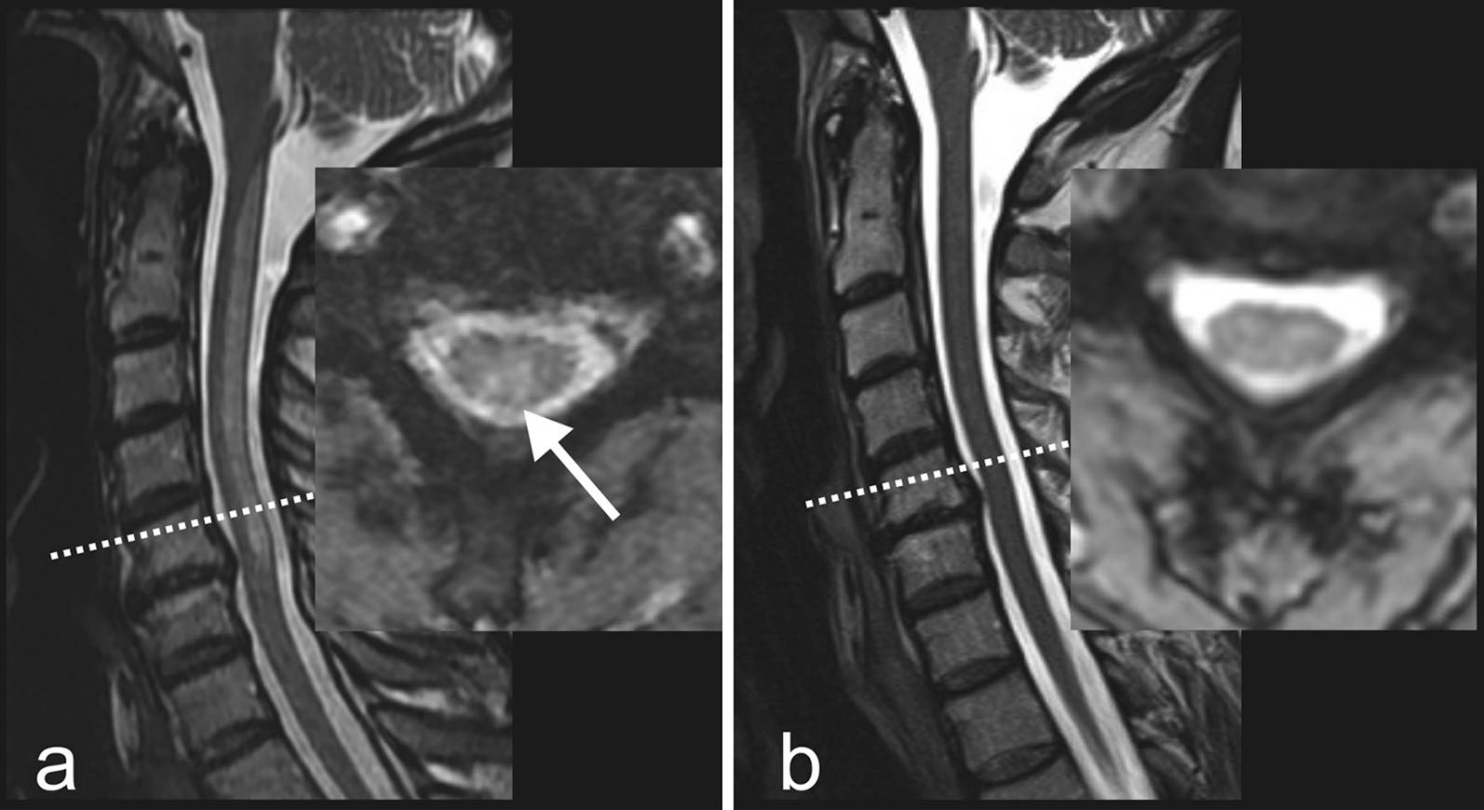
Magnetic resonance images of a patient suffering from subacute combined degeneration of spinal cord (SACD) before and after cobalamin substitution. **a** Before therapy: Sagittal and transversal T2-weighted images reveal an intraspinal hyper intensity of the dorsal cervical spinal cord (→) with no mass effect. No contrast enhancement of the lesion was found in T1-weighted images. **b** 5 months after the onset of cobalamin substitution: The hyper intensity completely disappeared. Known osteochondrosis and disc protrusion C5/6. In the sagittal view the transversal section plane is marked by a *dotted line*

The outward performed 3 Tesla MRI revealed a hyperintense T2w signal alteration in the dorsal cervical spine, corresponding to an intramedullar lesion reaching from the first to the fifth cervical vertebral body (Fig. 2a).

Motor evoked potentials (MEP) detected a marginal central-motoric latency to the left arm, indicating a potential pyramidal tract lesion. Somatosensory evoked potentials (SSEP) uncovered a medianus SSEP with marginal values on the left side, normal results on the right side and pathological values of the tibialis SSEP on both sides. Visual evoked potentials (VEP) showed prolonged latencies. In gastroscopy, the mucosa was atrophic. Histology of gastric biopsy revealed a chronic-atrophic inflammation of corpus ventriculi with a lack of intrinsic factor-producing gland cells and with a micronodular hyperplasia of neuroendocrine cells, indicating an autoimmune gastritis.

The laboratory diagnostic (Table 2) revealed elevated parietal cell antibodies (titer 1:640, normal: ≤1:20). Serum cobalamin concentration was in the lower normal range (197 pg/ml, normal: 150–900 pg/ml) and folic acid was normal (18.7 ng/ml, normal: 3–20 ng/ml), whereas MMA was highly elevated (40,800 nmol/l, normal: 50–300 nmol/l). In addition, we found an increased homocysteine of 50.8 µmol/l (normal: 5.0–15.0 µmol/l) and a high gastrin level (615 pg/ml, normal: 13–115 pg/ml). Blood cell count was normal. Further laboratory diagnostics did not reveal any renal dysfunction. Analysis of the cerebrospinal fluid revealed inconspicuous findings: a slight elevation of protein (530 mg/l, normal: <450 mg/l) and of lactate (2.4 mmol/l, normal: <1.9 mmol/l), an unremarkable cell count (1 cell/µl), and no oligoclonal bands.

**Table 2:** Laboratory values before and 11 month after cobalamin substitution

	Before cobalamin substitution	11 month after cobalamin substitution	Normal range
Cobalamin (pg/ml)	197	410	150–900
Homocysteine (µmol/l)	50.8	6.9	5.0–15.0
Methylmalonic acid (nmol/l)	40,800	178	50–300
Folic acid (ng/ml)	18.7	10	3–20
Gastrin (pg/ml)	615	730	13–115
Parietal cell antibodies	1:640	–	≤1:20

Despite distinct clinical findings of a subacute combined degeneration of spinal cord (SACD) and a profound intramedullar lesion shown by MRI, cobalamin was still in the normal range before therapy started. In contrast, homocysteine and methylmalonic acid are suitable parameters for SACD diagnostic. Methylmalonic acid is the most specific marker of a cobalamin deficiency. Homocysteine is suitable for follow-up and therapy monitoring. Just 5 month after therapy start clinical symptoms were declining and all pathological changes found by MRI in the spinal cord disappeared

### Therapy

**Fig. 3:**
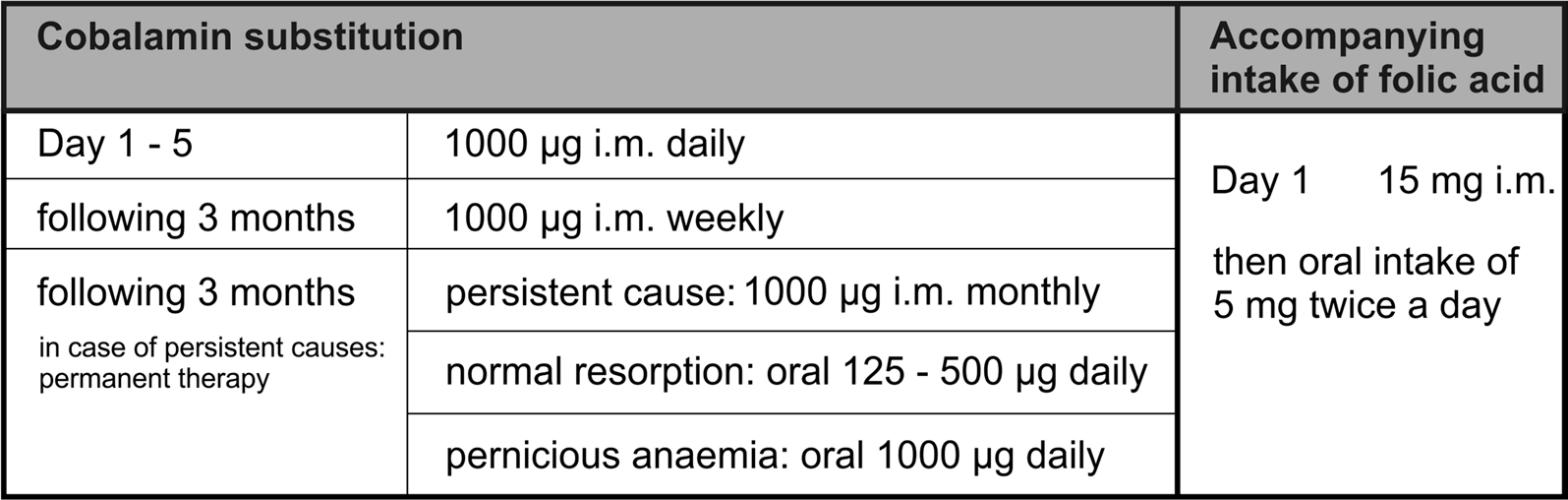
Treatment concept of subacute combined degeneration of spinal cord (SACD) as it is practiced in our clinic. The concept follows recommendations made by Herrmann et al. [7]. Early start of therapy is decisive for better treatment outcomes [5]

Our patient was treated with i.m. injections of 1000 µg cobalamin for 5 days. We advised a substitution of 1000 µg cobalamin i.m. once a week for the following 3 months, and afterward of 1000 µg/month i.m. Cobalamin substitution was accompanied by an intake of folic acid (Fig. 3).

### Outcome

Five months after the onset of therapy the patient complained of a symmetric hypaesthesia of both upper extremities, reaching from both hands to the shoulders. The hypaesthesia of the thorax had regressed. There were no other neurological problems and no behavioural abnormalities. At that time, serum level of cobalamin was 710 pg/ml (Table 2).

MRI of the cervical spine revealed a complete regression of the intraspinal myelon lesion (Fig. 2b). Electroneurography showed an axonal sensorimotor polyneuropathy of the upper and lower extremities.

After 11 months the patient reported paresthesia of both hands, the hypaesthesia of both arms had disappeared. Stereognosis of hands and feet was normal and two-point-discrimination was better (he recognized a distance of 4–5 cm at both arms and at the thorax). Bimalleolar vibration sense had improved from 0/8 to 4–5/8 as assessed by the scale of the vibration tune (0 no sense, 8 full vibration sense).

Gastrin level was still elevated (730 pg/ml, normal: 13–115 pg/ml), folic acid (10 ng/ml, normal: 3–20 ng/ml), cobalamin (410 pg/ml, normal: 150–900 pg/ml), homocysteine (6.9 µmol/l, normal: 5.0–15.0 µmol/l) and MMA (178 nmol/l, normal: 50–300 nmol/l) were of normal range (Table 2). Blood picture was unremarkable. MEP, SSEP and VEP revealed no abnormalities.

## Conclusions

Initially, the report of an accident three months before the onset of symptoms combined with a cervical myelon lesion suggested a traumatic injury. A neoplastic cause was considered for differential diagnosis. However, the lesion of the cervical spine cord only affected the dorsal part of the myelon without any mass effect and did not show any uptake of contrast agent. Furthermore, a subacute progress of disease was reported by the patient. Clinical examination revealed only sensory qualities conveyed by the posterior columns: hypaesthesia, reduction of two-point-discrimination, disturbed stereognosis, and reduced vibration sensation. Therefore, SACD became probable. Now, diagnosis had to be confirmed by laboratory tests. The patient had a cobalamin level in the lower normal range. Several publications described the determination of serum cobalamin to be unreliable [2, 3, 5, 18, 23, 24, 28, 29]. Therefore, other diagnostic markers are needed:

Adenosylcobalamin converts MMA to succinyl coenzyme A. Hence, cobalamin deficiency causes an excess of MMA [23]. Increased MMA values are highly sensitive and highly specific for cobalamin deficiency [28, 30].

For the degradation of homocysteine methylcobalamin, pyridoxine, and folic acid are needed. Therefore, hyperhomocysteinemia gives a hint of a deficiency of all these vitamins, and has a high sensitivity but low specificity for cobalamin deficiency [28, 30]. The homocysteine level is suitable for follow-up and therapy monitoring [31]. It needs to be considered that blood has to be cooled for determining homocysteine levels.

Finally, a deficit of cobalamin causes a reduction of holoTC [2, 23, 32]. Lowered serum holoTC concentration is the earliest marker of cobalamin deficiency and is reduced even before any clinical symptoms are apparent [2, 32].

In our case increased values for MMA and homocysteine were determined. After cobalamin substitution both values returned to normal. HoloTC was not measured, although we recommend its determination (Fig. 1).

The subsequent diagnostic provided prolonged latencies for the left-hand medianus and double-sided tibialis SSEP. Read in conjunction with the diagnostic imaging this can be explained by lesions in the fasciculus gracilis and cuneatus. MEP detected a central-motoric latency to the left arm pointing to an additional damage of the pyramidal tract. The elongated VEP give a hint for an undergoing demyelination of both optic nerves, which is sporadically associated with SACD. Nevertheless, our patient did not notice any visual limitations. In addition to the lesion in the cervical myelon, electroneurography revealed the existence of an axonal sensorimotor polyneuropathy, which is also common for a lack of cobalamin [33]. As expected in this context, liquor analysis did not reveal an infectious or chronic inflammatory process.

Gastroscopy and histology proved a type A gastritis with an increased value of gastrin. Serum gastrin is usually markedly increased as a result of gastric atrophy and the increase of pH value. Appropriately, parietal cell antibodies were found. Therefore, in this patient SACD was caused by a disruption of cobalamin processing in the stomach due to parietal cell antibodies inducing an increased pH-value and a decreased production of intrinsic-factor. Consequently, cobalamin could not dissolve out of the protein-bindings of the ingested food and was not bound to intrinsic factor.

Our patient presented solely sensory disturbances. No psychological disorders, no rhagades or gastrointestinal symptoms like Hunter glossitis, jaundice, diarrhea, dyspepsia or increased values of bilirubin were found. Moreover, no haematological alterations like macrocytosis were determined. A significant inverse correlation between the degree of anaemia and the severity of neurological involvement that was independent of the duration of symptoms is known [5, 27]. The reasons for this finding are unclear.

Therefore, it is important to think of SACD when only sensory disturbances can be found, even if the value of cobalamin is normal.

Vitamin substitution removed the lesion and nearly all clinical symptoms of cervical spine cord within 5 months and provoked a restitution of hypaesthesia of the thorax within eleven months. Therapy recommendations concerning dosage and mode of administration of cobalamin are inconsistent and depend on the underlying reason of vitamin deficiency [7, 24]. Figure 3 condenses our preferred treatment regime.

We reported on a patient with a large lesion in cervical spine cord that matched with a subacute combined degeneration of spinal cord (SACD). SACD was diagnosed by clinical signs and laboratory tests. When clinical signs suggest a lack of cobalamin, values of cobalamin might still be in the normal range. Therefore, it is important to determine more sensitive parameters: HoloTC is the earliest and MMA the most specific marker of a cobalamin deficiency. Measurement of homocysteine is inexpensive and therefore suitable for follow-up and therapy monitoring. Early and appropriate treatment reversed pathological changes in the spinal cord and dissolved associated clinical symptoms.

## Abbreviations

holoTC: holotranscobalamin
IF: intrinsic factor
MEP: motor evoked potentials
MMA: methylmalonic acid
SACD: subacute combined degeneration of spinal cord
SSEP: somatosensory evoked potentials
VEP: visual evoked potentials
